# RETRACTION: Effect of Retrieval Bags in Preventing Surgical Site Wound Infection During Elective Laparoscopic Cholecystectomy in Liver Cancer Patients: A Meta‐Analysis

**DOI:** 10.1111/iwj.70188

**Published:** 2025-01-12

## Abstract

**RETRACTION**: DingJ. , “Effect of Retrieval Bags in Preventing Surgical Site Wound Infection During Elective Laparoscopic Cholecystectomy in Liver Cancer Patients: A Meta‐Analysis,” International Wound Journal 20, no. 10 (2023): 4031–4039, 10.1111/iwj.14292.37424304 PMC10681484

The above article, published online on 10 July 2023, in Wiley Online Library (http://onlinelibrary.wiley.com/), has been retracted by agreement between the journal Editor in Chief, Professor Keith Harding; and John Wiley & Sons Ltd. Following an investigation by the publisher, all parties have concluded that this article was accepted solely on the basis of a compromised peer review process. The editors have therefore decided to retract the article. The authors did not respond to our notice regarding the retraction.

**RETRACTION**: J. Ding , “Effect of Retrieval Bags in Preventing Surgical Site Wound Infection During Elective Laparoscopic Cholecystectomy in Liver Cancer Patients: A Meta‐Analysis,” International Wound Journal 20, no. 10 (2023): 4031–4039, 10.1111/iwj.14292.37424304 PMC10681484

The above article, published online on 10 July 2023, in Wiley Online Library (http://onlinelibrary.wiley.com/), has been retracted by agreement between the journal Editor in Chief, Professor Keith Harding; and John Wiley & Sons Ltd. Following an investigation by the publisher, all parties have concluded that this article was accepted solely on the basis of a compromised peer review process. The editors have therefore decided to retract the article. The authors did not respond to our notice regarding the retraction.

